# ESBL‐production in *Escherichia coli *and *Klebsiella pneumoniae *isolates from Nigeria

**DOI:** 10.1002/mbo3.816

**Published:** 2019-02-22

**Authors:** Frederik B. Hertz, Fillip Jansåker, Kenneth O. Okon, Ibrahim S. Abdulmumin, Joseph O. Onah, Joshua Ladan, Jenny D. Knudsen

**Affiliations:** ^1^ Department of Clinical Microbiology, Hvidovre Hospital University of Copenhagen Hvidovre Denmark; ^2^ Department of Virus & Microbiological Special Diagnostics Statens Serum Institut Copenhagen Denmark; ^3^ Department of Medical Microbiology, Federal Medical Centre Makurdi Nigeria; ^4^ Clinton Health Access Initiative Abuja Nigeria; ^5^ Department of Disease and Immunization National Primary Health Care Development Agency Abuja Nigeria; ^6^ Department of Medical Microbiology Abubakar Tafawa Balewa University Teaching Hospital Bauchi

**Keywords:** Enterobacteriaceae, ESBL, *Escherichia coli*, *Klebsiella pneumoniae*, Nigeria

## Abstract

The worldwide increase in infections caused by extended‐spectrum beta‐lactamase‐ (ESBL) and AmpC‐producing Enterobacteriaceae (ESBL‐E) is a concern. Surveillance is extensive in Europe, North America, and Asia. Yet, there is no summarizing surveillance in Africa. This study aimed to perform a preliminary investigation on the prevalence of ESBL‐E in the northeastern part of Nigeria. However, of the 60 samples collected, we were able to culture 15 *Escherichia coli* and 7 *Klebsiella *spp. only. In the collection of clinical hospital samples, we found eight of 15 *E. coli *isolates to be ESBL (53%) and two out of seven *Klebsiella *spp. to be ESBL/AmpC (29%). Due to the limitations of this study, our findings cannot take a broad view on the prevalence of ESBL‐E, in Nigeria and other parts of Africa. Yet, to know which genes encode ESBL in Nigeria, and to know exact prevalence of every ESBL gene would be of importance.

## COMMENTARY

The worldwide increase in infections caused by extended‐spectrum beta‐lactamase‐ (ESBL) and AmpC‐producing Enterobacteriaceae (ESBL‐E) has been a concern. Surveillance is extensive in Europe, North America, and Asia. Yet, there is no summarizing research or surveillance in Africa (Storberg, 2014). In studies from Africa, ESBL‐E has logically been found to vary between countries, but found to be common (Storberg, 2014). A recent publication by Elikwu et al., (2017) on etiology and antimicrobial susceptibility patterns from urinary tract infections in Nigeria described that of all bacteria isolated from urine samples only 4% were caused by ESBL. Denmark has similar prevalence rate and considered a low‐prevalence country (Pedersen, 2016; Storberg, 2014). Furthermore, almost 30% of *Escherichia coli* in the study were found to be resistant to cefpodoxime, a drug often used to screen for the presence of ESBL (Elikwu et al., 2017). Therefore, the rate of ESBL might be much higher than previously reported.

This study aimed to investigate presence of phenotypic ESBL‐production in *E. coli *and *Klebsiella pneumoniae *isolates from the northeastern part of Nigeria. This was performed as a collaboration between health care professionals in Bauchi, Nigeria and Hvidovre Hospital (HVH) Copenhagen, Denmark. Sixty clinical isolates of *E. coli *or *Klebsiella *spp. from a biobank collected in 2015 in Bauchi, Nigeria, were sent to Dept. of Clinical Microbiology at HVH in Denmark. The origin for the isolates were urine and blood. All *E. coli *and *Klebsiella *spp. were originally identified in Nigeria and stored at −80°C. Samples were shipped to Denmark in transport media (Nutrient agar, SLOPE) without temperature control. At HVH, Denmark, all samples were subcultured on Mueller–Hinton agar plates as well as a enteric blue plate (SSI Diagnostica A/S, Hillerød, Denmark). Identification of bacteria cultures on agar plates was done at the species level by matrix‐assisted laser desorption–time of flight analysis (MALDI‐TOF) (Bruker, Germany). Antimicrobial susceptibility testing was performed by the disk diffusion test methodology as described in the European Committee on Antimicrobial Susceptibility Testing (EUCAST, Version 1.0, 18 December 2009) and interpreted according to EUCAST. We tested a variety of antibiotics typically used to treat infections caused by *E. coli *and *K. pneumoniae *(gentamicin, ampicillin, piperacillin/tazobactam, nalidixic acid, ciprofloxacin, ciprofloxacin, meropenem, mecillinam, amoxicillin/clavulanic acid, cefpodoxime, nitrofurantoin, trimethoprim, and sulfamethizol). Strains with a zone diameter for cefpodoxime of ≤24 mm (Oxoid disks) were ESBL‐screening test‐positive. ESBL‐ or AmpC production was phenotypically confirmed by the MAST test, performed as a combined‐disk method, using disks containing cefpodoxime ±ESBL and/or AmpC inhibitors (MAST®, Merseyside, UK) (Hertz et al., 2015).

**Figure 1:**
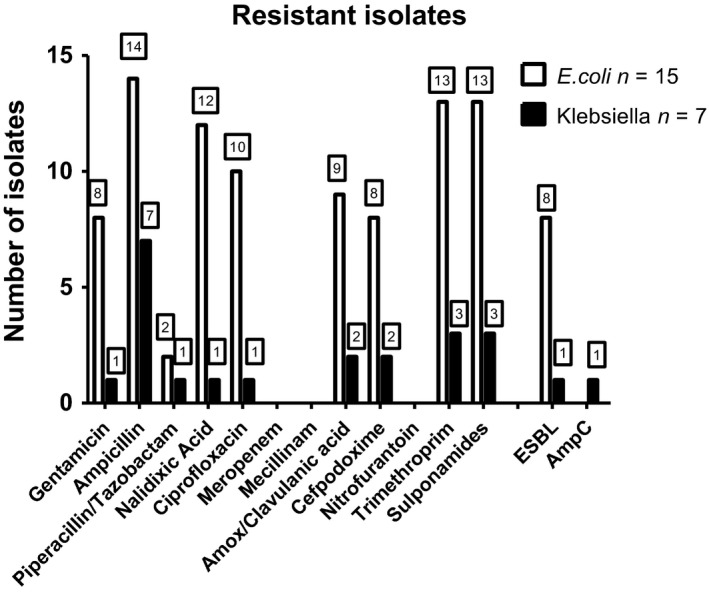
Resistance pattern for the 22 samples found as monoculture of *Escherichia coli *or *Klebsiella *spp*. *Here, the pathogens were considered to be the true pathogen. The number shown is the number of isolates resistant to the antibiotic. Eight *E. coli *isolates were found to be ESBL producing and two *Klebsiella pneumoniae *isolates were ESBL and AmpC producing, respectively. ESBL: extended‐spectrum beta‐lactamase

Of the 60 samples collected, one was without any bacterial growth. In the remaining 59 samples, we were able to culture 15 *E. coli* and 7 *Klebsiella *spp. (6 *K. pneumoniae* and 1 *Klebsiella oxytoca*) only. Results of antibiotic susceptibility testing are found in Figure 1. Of the 15 identified *E. coli*, eight produced ESBL, but none produced AmpC. The majority of *E. coli *were resistant to trimethoprim (*n* = 13), sulfamethizol (*n* = 13), and ciprofloxacin (*n* = 10), yet none were resistant to mecillinam (*n* = 0) or nitrofurantoin (*n* = 0) (Figure 1). Of the seven *Klebsiella *spp., one produced ESBL and one produced AmpC. Besides resistance to ampicillin, which is intrinsic in *K. pneumoniae *and *K. oxytoca,* resistance was low among *Klebsiella *spp. None of the isolates were found to be carbapenemase producers.

Thus, in the collection of clinical hospital samples from Bauchi, Nigeria, we found eight of 15 *E. coli *isolates to be ESBL (53%) and two out of seven *Klebsiella *spp. to be ESBL/AmpC (29%).

Our study has several limitations. First, all bacterial cultures were transferred from Nigeria to Denmark without temperature control and most of these cultures were therefore contaminated. Second, only 22 bacterial isolates were left why we cannot generalize on the prevalence of ESBL‐ and AmpC‐producing Enterobacteriaceae in Nigeria. Third, we have not considered selection bias.

Due to the limitations of this study, our findings cannot take a broad view on the prevalence of ESBL‐E in Nigeria and other parts of Africa. Yet, to know which genes encode ESBL in Nigeria, and to know exact prevalence of every ESBL gene would be of importance. Such a study must take identification of isolates in Nigeria and subsequent transportation of isolates into consideration.

## CONSENT FOR PUBLICATION

All authors have read the data note and agreed to it being submitted for publication.

## CONFLICT OF INTEREST

None declared.

## AUTHORS CONTRIBUTION

All authors meet the authorship criteria, and nobody who qualifies for authorship has been omitted from the list; authors have approved the acknowledgement of their contributions. All authors contributed to the study design, interpretation of the data, intellectual discussion and/or revision of the data note. FBH, JDK, and KOO participated in the planning of laboratory research and were responsible for the original study idea and study design. ISA, JOO, and JL performed the sample collection. FJ helped with collaboration between our countries. FBH wrote the initial draft of the data note.

## ETHICS STATEMENT

All patients in this nonintervention study were treated with care by healthcare professionals, in line with all other patients with similar infections and according to current guidelines. No further information from patients was obtained. The project did not involve any health risks and was in no way at the expense of the subject. All data were anonymized. Thus, no ethical approval or consent was necessary.

## Data Availability

All data associated with the study is presented in the article.
